# SF-36 predicts 13-year CHD incidence in a middle-aged Swedish general population

**DOI:** 10.1007/s11136-019-02362-y

**Published:** 2019-11-13

**Authors:** Evalill Nilsson, Karin Festin, Mats Lowén, Margareta Kristenson

**Affiliations:** 1 Department of Medical and Health Sciences, Linköping University, Linköping, Sweden; 2 Department of Health and Care Development, Linköping University, Linköping, Sweden

**Keywords:** Health-related quality of life, Morbidity, Mortality, Heart disease prevention

## Abstract

**Purpose:**

To study the predictive ability of each of the eight scales of SF-36 on 13-year all-cause mortality and incident coronary heart disease (CHD) in a general middle-aged population.

**Methods:**

The population-based, longitudinal “Life-conditions, Stress and Health” study, in 2003–2004 enrolled 1007 persons aged 45–69 years (50% female), randomly sampled from the general population in Östergötland, Sweden. Variables at baseline included the SF-36 (health-related quality of life, HRQoL) and self-reported disease. Incident CHD (morbidity and mortality) and all-cause mortality data for the study population during the first 13 years from baseline were obtained from national Swedish registries.

**Results:**

Seven of the eight SF-36 scales predicted CHD (sex- and age-adjusted Hazard Ratios up to 2.15; *p* ≤ 0.05), while only the Physical Functioning scale significantly predicted all-cause mortality. Further adjustments for presence of (self-reported) disease did not, in most cases, alter these significant predictions.

**Conclusion:**

Low SF-36 scores predict risk of CHD, also after adjustment for present disease, supporting the biopsychosocial model of health and disease. Measures of HRQoL yield important information and can add to the cardiopreventive toolbox, including primary prevention efforts, as it is such a simple and relatively inexpensive tool.

## Introduction

The use of questionnaires on health-related quality of life (HRQoL) is nowadays commonplace in population surveys and as patient-reported outcome measures (PROMs) in health care. One of the first widespread HRQoL questionnaires was the SF-36, a generic measure with eight scales, sometimes clustered in two component summary scores: physical (PCS) and mental (MCS) [1]. The SF-36 has been used both in general populations and in many different patient groups.

Though mostly used as a measure of present health status or outcome, the SF-36 has also been investigated for its ability to predict future health outcomes (including mortality) and healthcare use. Mostly, these study populations have included different patient groups, but both general populations and more specific populations, such as veterans, have also been studied [2–8]. However, most of them included only elderly people. In addition, most studies have focused on investigating the two summary scores. The main finding seems to indicate an association with mortality, with low scores on the PCS (more often) and MCS (more seldom) predicting future all-cause mortality (1–15 years follow-ups) [2–8]. In addition, low scores on the Physical functioning scale alone have been shown to predict all-cause mortality [4, 7].

While incidence of coronary heart disease (CHD) has fallen dramatically in high-income countries, it is still the leading cause of death [9]. Thus, we still need to know more about causes, risk factors and predictors of incident CHD. The aim of the present study is to investigate the predictive power of SF-36 (all eight scales) on CHD incidence and all-cause mortality in a Swedish general middle-aged population.

## Methods

### Study population

The longitudinal study “Life-conditions, Stress and Health” (LSH) was designed to investigate to what extent psychosocial factors can explain socioeconomic differences in risk of CHD, and if psychobiological pathways can mediate these associations. Participants were randomly selected from the general population and invited consecutively to reach a study population size of *n* = 1000, evenly distributed by age and sex. All citizens in the given age-range living in the catchment areas of ten primary health care centres in the County of Östergötland in South East Sweden at the time of enrolment were eligible for invitation. Participation rate was 62.5%, resulting in 502 males and 505 females, aged 45–69 years.

Participants visited their primary health care centres in late 2003 and early 2004 for a brief health examination, and at baseline several health questionnaires were answered. The sample was representative of the population in terms of educational attainment, employment rate and immigrant status [10].

### SF-36

The Swedish standard version of SF-36 was used to measure HRQoL at baseline, with a higher score indicating a better HRQoL [11]. All but the second of the 36 items in the SF-36 are aggregated into eight multi-item scales: physical functioning (PF), role physical (RP), bodily pain (BP), general health (GH), vitality (VT), social functioning (SF), role emotional (RE) and mental health (MH). In the present study the 0 to 100 scoring algorithm was used. As we aimed for a deeper understanding on effects of each scale, we were not interested in the component summary scores.

### 13-year incident CHD and all-cause mortality

Incident CHD was defined as first-time fatal or nonfatal myocardial infarction and/or an event of symptomatic angina pectoris requiring invasive coronary revascularisation (percutaneous cardiac intervention or coronary artery by-pass graft-surgery). CHD and all-cause mortality data for the LSH study population during the first 13 years from baseline (2004–2016) were obtained from the National Causes of Death Register and the National Patient Register, both sourced from the Swedish National Board of Health and Welfare.

### Self-reported disease

Besides sex and age, presence of disease is associated with both exposure and outcome, and this impact needs to be considered. The baseline questionnaire asked whether the individual had, by a physician, been diagnosed with any of the listed common medical conditions (myocardial infarction, stroke, angina pectoris, cancer, diabetes, chronic obstructive pulmonary disease, rheumatic disease, asthma/allergy, gastrointestinal disease, diseases in muscles and joints, neurological diseases, other disease), and they were asked whether they had experienced back and/or neck pain (check boxes). Data were dichotomised into ‘yes’ (having been diagnosed with at least one of the diseases in the list, and/or having back and/or neck pain) or ‘no’ regarding having a disease (or diseases).

### Data analysis

Baseline SF-36 data were categorized into tertiles for five of the scales (PF, BP, GH, VT and MH). Due to skew distributions, the three role functioning scales (SF, RE and RP) were only dichotomised into high and low.

Cox proportional regression models were used to investigate the association between each of the eight scales of SF-36 at baseline and two separate outcome measures at follow-up 13 years later: first event of incident CHD (excluding 20 persons with previous myocardial infarction) and all-cause mortality during the 13-year follow-up period. The models were carried out in two steps: first adjustment for age and sex, then additionally for self-reported diagnoses and experience of back and/or neck pain at baseline. Schoenfeld residual analysis and deviance residuals analysis were used to evaluate the models.

## Results

CHD constituted 15% of the deaths in the study population during the 13-year period (Table 1). SF-36 values at baseline were similar to earlier Swedish general population studies [11].

**Table 1:** Study population characteristics

	Total study population (*n* = 1001)
Sex, female (%)	50
Age, mean ± SD (years)	57 ± 7
Self-reported disease or back pain (*n*, %)	415, 51
CHD, 13-year follow-up (*n*, %)	94, 9.1
Mortality, all-cause, 13-year follow-up (*n*, %)	111, 11.1
Mortality, CHD, 13-year follow-up (*n*, %)	17, 1.0
SF-36 (941 ≤ *n* ≤ 971)	
Physical functioning, mean ± SD	84 ± 18
Role physical, mean ± SD	80 ± 34
Bodily pain, mean ± SD	69 ± 26
General health, mean ± SD	70 ± 21
Vitality, mean ± SD	66 ± 23
Social functioning, mean ± SD	87 ± 20
Role emotional, mean ± SD	86 ± 30
Mental health, mean ± SD	79 ± 18

Baseline data unless otherwise stated

*CHD* coronary heart disease

In analyses of 13-year risk of a first CHD event, after adjustment for age and sex, significant associations exist for all scales of the SF-36, when comparing t1 to t3, except for Role Emotional (*p*-value marginally above 0.05) (Table 2). After control for presence of somatic disease (Model 2), associations remain for most of the SF-36 scales. No significant differences in risk of MI between t1 and t2 were identified. Schoenfeld residual analysis confirmed that the proportional hazard assumption was met. Deviance residuals were generally centred about zero with no obvious trend.

**Table 2:** Associations between the eight scales of the SF-36 (at baseline) and 13-year risk of a first CHD event as well as all-cause mortality

	CHD				All-cause mortality			
	Model 1^a^		Model 2^b^		Model 1^a^		Model 2^b^	
	Hazard ratio (95% CI)	p*	Hazard ratio (95% CI)	p*	Hazard ratio (95% CI)	p*	Hazard ratio (95% CI)	p*
Physical functioning		**0.043**		0.186		0.073		**0.028**
t1 versus t3^c^	1.69 (1.03–2.76)	**0.037**	1.37 (0.80–2.33)	0.248	1.42 (0.91–2.23)	0.123	1.66 (1.02–2.70)	**0.040**
Role physical	1.86 (1.19–2.91)	**0.006**	1.67 (1.05–2.65)	**0.029**	1.34 (0.89–2.02)	0.162	1.40 (0.92–2.13)	0.120
Bodily pain		**0.012**		0.114		0.869		0.568
t1 versus t3	2.14 (1.29–3.53)	**0.003**	1.84 (1.03–3.29)	**0.038**	1.07 (0.70–1.64)	0.745	1.27 (0.78–2.08)	0.336
General health		**0.015**		0.105		0.124		0.063
t1 versus t3	2.15 (1.26–3.66)	**0.005**	1.82 (1.04–3.17)	**0.035**	1.19 (0.77–1.84)	0.425	1.33 (0.84–2.10)	0.223
Vitality		0.053		0.232		0.380		0.348
t1 versus t3	1.67 (1.02–2.74)	**0.044**	1.37 (0.81–2.31)	0.246	1.07 (0.66–1.73)	0.790	1.16 (0.70–1.92)	0.566
Social functioning	1.80 (1.17–2.76)	**0.007**	1.65 (1.07–2.56)	**0.025**	1.12 (0.80–1.67)	0.560	1.17 (0.79–1.76)	0.431
Role emotional	1.64 (0.99–2.71)	0.056	1.51 (0.91–2.51)	0.104	0.97 (0.58–1.60)	0.896	0.99 (0.60–1.64)	0.965
Mental health		**0.007**		**0.022**		0.640		0.543
t1 versus t3	1.64 (1.01–2.67)	**0.044**	1.43 (0.87–2.34)	0.162	1.22 (0.76–1.94)	0.413	1.28 (0.79–2.06)	0.317

Bold values indicate statistically significant *p* < 0.05

*p-value indicating (1) *p*-value for the overall effect the specific scale has on risk of a first CHD (coronary heart disease) event for scales with more than two categories, and (2) *p*-value for the dummies when compared to the reference category, i.e. t3 compared to t1

^a^Adjusted for age and sex

^b^Adjusted for age, sex and reporting at least one disease and/or neck/back pain

^c^Tertile 1 (t1) versus tertile 3 (t3). Differences between t1 and t2 were not significant and therefore not presented in the table

For 13-year risk of all-cause mortality the only significant association found was for the PF scale between t1 and t3, and only when adjusting for presence of disease.

## Discussion

This is, to our knowledge, the first study to examine the predictive power of each of the eight scales of the SF-36 on incident CHD risk in a general middle-aged population. We found that in this population most SF-36 scales were predictive of first-time CHD (HR 1.5–2.1). In contrast, only the PF scale was predictive of all-cause mortality.

Regarding the predictive ability of SF-36 for CHD morbidity, there are some studies on patients with, for example, heart failure and diabetes. These studies show that low scores on SF-36 is predictive of future coronary events [12, 13]. Studies regarding the predictive power of the scales of SF-36 on incident CHD in a middle-aged general population are scarce. The theoretical basis for a relationship between HRQoL and disease outcome can be traced back to earlier studies on causes of the predictive effect of self-reported health (SRH), for which several possible interpretations have been offered. SRH may capture sub-clinical or undiagnosed disease, may influence health behaviours, modify the effects of biomedical risk factors or reflect the presence or absence of psychosocial risk factors or resources [14]. We earlier reported (with data from this cohort) a significant impact of psychological factors on SF-36 results [15] and a relationship between low HRQoL and low-grade sub-clinical inflammation [16].

When adjusting for presence of somatic disease, the effect persists for most of the scales (not PF and VT), although the hazard ratio is below two. This finding is concordant with, and supports, the notion that measures of HRQoL, regarding how we perceive our physical and social functioning, are not merely dependent on presence of disease and absolute functional ability, but also on psychosocial risk factors or resources, e.g. mood and confidence [15, 17], i.e. the biopsychosocial model [18].

We also could reproduce findings from earlier studies where PF predicted all-cause mortality. None of the other scales of SF-36 was related to risk of all-cause mortality in the present study, but as mentioned, the predictive value of the individual scales is not well studied. Moreover, incident CHD is today becoming less fatal. This reduction in CHD mortality might be part of the explanation as to why the predictive ability of HRQoL on (all-cause) mortality seems not to be as strong as it was previously.

### Methodological issues and further research

The finding that not all eight scales show significant associations with cardiac events could, given that *p*-values are just above 0.05, possibly be due to the population size (power) and the construction of the different scales, rather than due to some domains not being predictive. Furthermore, the present study was not designed to explain the identified predictive effect. Future research should use a larger population to further explore the predictive effect, e.g. individual effects of different diseases and a broader set of confounders. Threshold modelling may be used to better understand the association between score on the SF-36 scales and risk of MI or all-cause mortality.

## Conclusion

Low SF-36 scores predict risk of incident CHD, also after adjustment for present disease, supporting the biopsychosocial model of disease. Measures of HRQoL yield important information and could add to the cardiopreventive toolbox, including primary prevention efforts, as it is such a simple and relatively inexpensive tool.
